# How prism adaptation reveals the distinct use of size and positions in grasping

**DOI:** 10.1007/s00221-022-06506-4

**Published:** 2022-11-12

**Authors:** Jeroen B. J. Smeets, Ian Pennekamp, Bente van Amsterdam, Willemijn D. Schot

**Affiliations:** 1 Department of Human Movement Sciences, Vrije Universiteit Amsterdam, Van der Boechorststraat 9, NL-1081 BT Amsterdam, The Netherlands; 2 Educational Development and Training, Utrecht University, Utrecht, The Netherlands

**Keywords:** Sensorimotor adaptation, Prehension, Inconsistent perception, Goal-directed movement

## Abstract

The size of an object equals the distance between the positions of its opposite edges. However, human sensory processing for perceiving positions differs from that for perceiving size. Which of these two information sources is used to control grip aperture? In this paper, we answer this question by prism adaptation of single-digit movements of the index finger and thumb. We previously showed that it is possible to adapt the index finger and thumb in opposite directions and that this adaptation induces an aftereffect in grip aperture in grasping. This finding suggests that grasping is based on the perceived positions of the contact points. However, it might be compatible with grasping being controlled based on size provided that the opposing prism adaptation leads to changes in visually perceived size or proprioception of hand opening. In that case, one would predict a similar aftereffect in manually indicating the perceived size. In contrast, if grasping is controlled based on information about the positions of the edges, the aftereffect in grasping is due to altered position information, so one would predict no aftereffect in manually indicating the perceived size. Our present experiment shows that there was no aftereffect in manually indicating perceived size. We conclude that grip aperture during grasping is based on perceived positions rather than on perceived size.

## Introduction

Since the pioneering experiment of Aglioti et al. (1995) comparing the effect of the Ebbinghaus illusion on perception and grasping, many authors have been puzzled by the question why visual illusions seem to have less effect on grip aperture during grasping than one might have expected given the perceptual effect. Interestingly, this paper is frequently cited as providing evidence that grasping is immune to the illusion, although the data (Fig. 5 of that paper) show that the effect on grip aperture was about 65% of the effect on perceptual matching. Should we interpret this finding indeed as an indication that grasping is immune to the effect of contextual visual illusions or not?

One way to interpret the smaller effect on grip aperture than on perceptual judgements is that these two effects have the same magnitude, but the details of the design and analysis are the cause of an apparent difference. Two aspects play a role here: the design of the perceptual task and the analysis of kinematic data. Firstly, several studies showed that the perceptual effect is overestimated by Aglioti et al. (1995) because their very elegant design of the judgement task is based on the comparison of two disks in contrasting contexts, whereas the effect on grip aperture can be regarded as being due to a single context (Pavani et al. 1999; Franz et al. 2000). Indeed, the perceptual effect reduces to the magnitude of the effect on peak grip aperture if a different design of the perceptual task is used. Secondly, the effect on grip aperture is underestimated because grip aperture is not a fixed safety margin larger than object size, but increases with a fraction of about 80% of the increase in object size (Smeets and Brenner 1999). For a fair comparison, the effect on grip aperture should thus be corrected for this fraction (Hesse et al. 2016a).

The alternative interpretation is that the grip aperture is immune to the size illusions because the observed effect is not due to the size illusion, but other aspects of the stimulus. Two lines of reasoning lead to this interpretation. The first line of reasoning argues that the effect may emerge because the context items that induce the Ebbinghaus illusion act as obstacles (Haffenden et al. 2001), even though they were only drawn. This reasoning is in line with other experiments showing that context items that do not physically interfere can indeed act as obstacles. For instance, rotation of such context items around the centre of the object makes participants choose a different grip orientation when grasping that object (de Grave et al. 2005). However, careful experimentation has shown that this obstacle-avoidance effect cannot explain the effect of the Ebbinghaus illusion on grip aperture (Franz et al. 2003; Kopiske et al. 2016). This evidence, however, does not refute the alternative explanation, as it is supported by the second line of reasoning, based on the notion that our perception is not necessarily consistent (Smeets et al. 2002; Sousa et al. 2011; Smeets and Brenner 2019).

The second line of reasoning that interprets grip aperture as being immune to size illusions starts with the observation that the perceived size of an object is not necessarily consistent with the perceived position of the edges of that object (Smeets et al. 2002; Smeets and Brenner 2019). It combines this notion of inconsistencies in perception with the finding that there is not a single consistent effect of illusions on all aspects of an action. Although the Ponzo illusion does not affect maximum grip aperture, it clearly influences initial lift force (Brenner and Smeets 1996) and grip force (Jackson and Shaw 2000). Based on this finding, we have argued that the prevalent view that grip formation is based on processing size information (Jeannerod 1999) is incorrect. Instead, we have proposed a new view on grasping in which the grip aperture emerges from the control of the position of the digits’ tips in space based on location information (Smeets and Brenner 1999). In the 20 years after this proposal, we have provided a wide range of experimental results supporting this digit-in-space hypothesis (reviewed by Smeets et al. 2019). The reason why all the authors find an effect of the Ebbinghaus illusion on grip aperture is that this illusion not only affects the perceived size but also perceived positions (unlike many other size illusions; Smeets and Brenner 2019). For illusions that only affect perceived size, there is no effect of the illusion on peak grip aperture (Smeets et al. 2020). However, the digit-in-space hypothesis (the idea that hand shaping during the reach-to-grasp movement is controlled based on position information, rather than on size information) seems a priori very unlikely. We thus need a strong and direct test of this hypothesis.

A popular way to test the nature of motor control is to test how learning generalises (Shadmehr 2004; Berniker and Körding 2008). The digit-in-space hypothesis assumes that the index finger and thumb are controlled independently despite sharing the same arm muscles. If so, it should be possible to adapt the two digits independently to a new mapping of visual positions. To test this, we let participants touch an object with either the thumb or the index finger (Schot et al. 2014). For trials with the thumb, they viewed the object through a prism that shifted positions leftwards; for trials with the index finger, they viewed it through a prism that shifted positions rightwards (and an opposite pairing in a second session). Participants could adapt the two digits simultaneously to these opposite prism displacements. If the grip formation in grasping is indeed based on the separate control of the finger and thumb, this adaptation should transfer to the grip aperture, which it indeed did (Schot et al. 2017). Although this pattern of generalisation is what one would predict for the digit-in-space hypothesis, we did not experimentally rule out alternative explanations for this transfer. For instance, it might be the case that the opposing prism adaptation leads to changes in perceived visual size or in changes in proprioception of hand shape, which would provide an alternative explanation for the observed generalisation to grip aperture.

For the present paper, we question whether the opposite adaptation of the index finger and thumb in a touching task leads to a change in how one shapes one’s hand to match the size of an object. We thus asked participants to indicate the size of an object by opening their hand until the distance between the finger and thumb matched their percept. This task is a perceptual task that resembles best the grasping task (Pavani et al. 1999; Franz et al. 2000), as participants indicate their percept of size using the same effectors as they use in grasping. It has been shown that indicating the perceived size in this way results in similar estimates as more conventional psychophysical methods to assess the perception of size (Franz 2003). Most importantly, whether we will find that adaptation of the individual digits induces an aftereffect or not in the hand opening in this perceptual task will provide an answer to the long-debated question whether grip aperture in grasping is based on the same size information as perceptual judgements (Schenk et al. 2011; Kopiske et al. 2016) or based on perceived locations (Smeets et al. 2019, 2020).

## Methods

### Participants

Twelve volunteers (6 male, 6 female; aged 19–21 years) participated in the study. We based the number of participants on our previous experience in a similar paradigm (Schot et al. 2017). To prevent a situation in which finding no effect on grip aperture in this experiment (in contrast to the significant effect of Schot et al. 2017) might be due to lack of power, we decided to use 12 instead of 8 participants. All participants were right-handed and used this hand in the study. They had no known neurological deficits and normal or corrected-to-normal vision. The study is part of a program that has been approved by the ethical committee of Behavioural and Movement Sciences of the Vrije Universiteit Amsterdam.

### Setup and procedure

**Fig. 1:**
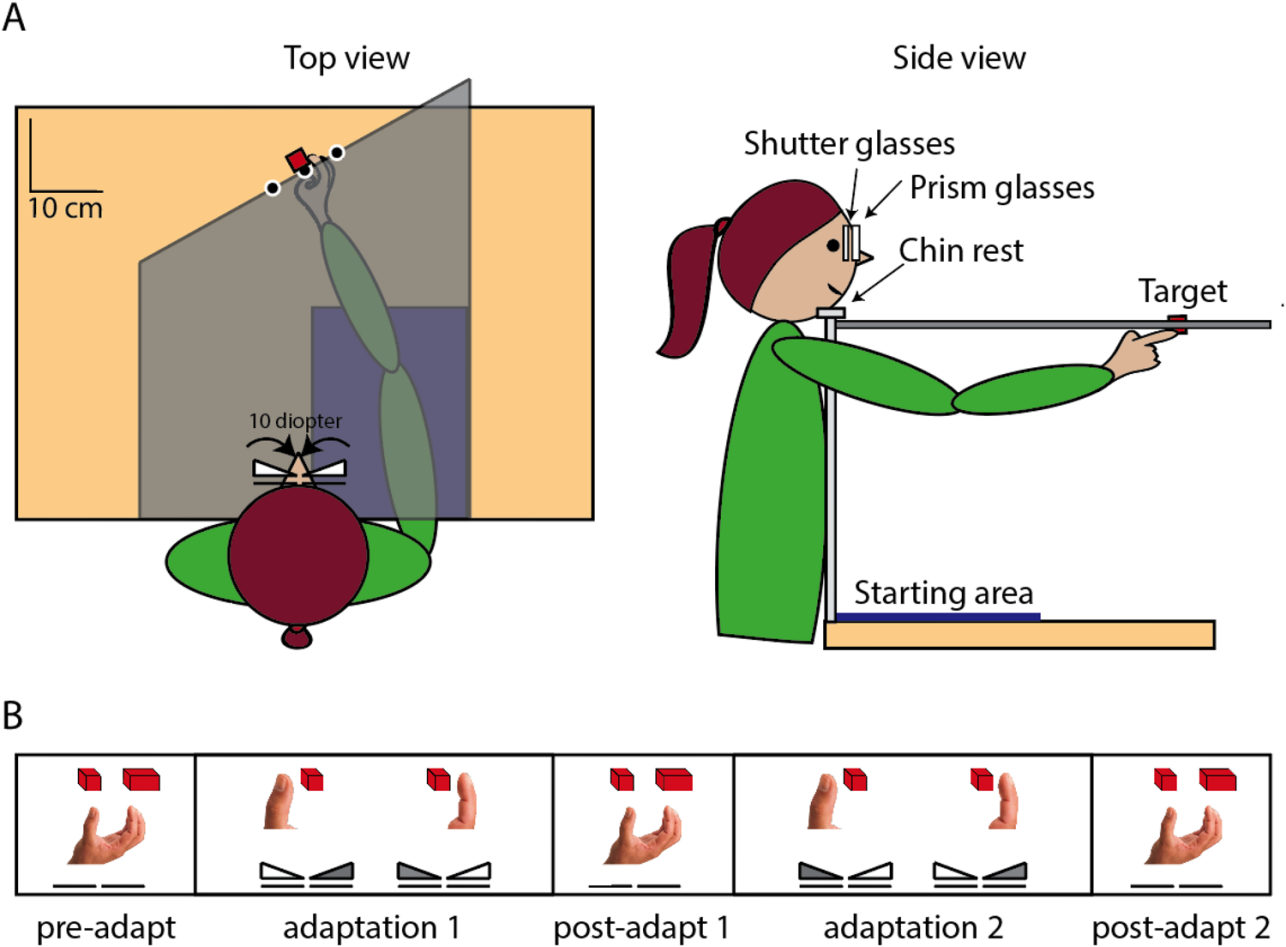
Methods. **A** The experimental setup with a participant touching the target cube with the index finger during one of the adaptation phases. The grey board that prevented vision of the arm is in this figure made transparent for illustrative purposes. **B** The five phases of the experiment. During the adaptation phases, the participants viewed the target monocularly through one of the prisms and touched it with either the index finger or the thumb. During the other phases, participants manually indicated the size of the target they viewed binocularly

Our experiment uses to a large extent the same setup and the same procedure as was used by Schot et al. (2017). To make explicit that a few aspects were different, we mention these differences in the description below. The position of infrared emitting diodes attached to the fingernails of the thumb and index finger were recorded at 250 Hz using an Optotrak 3020 system. The diodes were attached to the fingernails to optimise visibility when indicating size, rather than movements towards the cube. To be able to check whether participants responded to size, we used two objects: a target cube (about 2.5 cm) and a twice as large target cuboid. In each trial, one target object was attached to a wooden board at one of three possible target locations (5 cm apart, about 45 cm from the eyes of the participant, see Fig. 1A). We used multiple target locations to ensure that participants made new visuomotor transformations in each trial, so that adaptation would generalise. We used a board to prevent participants’ vision of the hand; the far edge was oriented at 30° to let the orientation of the cube match the preferred hand orientation (Schot et al. 2010). The board was wide and covered with a uniformly grey foil to prevent the use of visual references. Participants started and finished all trials with their right hand with the palm down and fingers spread on an A4-sized starting area of corrugated cardboard. We decided not to use the starting cube that Schot et al. (2017) used because holding such a cube after each trial would provide a recalibration of haptic size estimates. We asked participants to put their left hand in a comfortable position away from their right hand. To ensure similar posture and viewing angle across the whole experiment, we provided a chinrest at the level of the board.

To induce prism adaptation, we asked participants to touch the side of the target object with a single digit while viewing the target object through a 10-diopter prism (VTE Yoked Rotating Prisms, Bernell, Mishawaka, IN, USA). To induce adaptation in opposite directions for the index finger and the thumb, we used two different orientations of the prism, so that whether the prism deviated the optical location to the left or the right depended on the digit that was used in that trial. To be able to switch between prism orientation quickly, we let participants view the target monocularly (using PLATO shutter glasses) with prisms in front of both eyes. These two prisms were oriented in opposite directions, so viewing with the left eye resulted in a 5 cm rightward target deviation and viewing with the right eye resulted in a 5 cm leftward target deviation.

The experiment was executed as a single session that consisted of five phases: one pre-adaptation phase, two adaptation phases, and two post-adaptation phases. We decided to combine the two adaptation phases in a single session (combining the two separate sessions of Schot et al. 2017) to reduce variability. In all phases, each trial started and ended with the glasses in front of both eyes switched to opaque to let the experimenter switch the target. The pre- and post-adaptation phases each consisted of 30 size indication trials, in which we presented each combination of the two target sizes and three target positions five times. In these phases of the experiment, we provided participants with unperturbed binocular vision by opening the PLATO glasses in front of both eyes at the start of each trial. We asked participants to indicate the size by lifting their invisible right hand from the cardboard and indicate the size of the object by opening the hand accordingly and keeping it open for 2 s.

Each adaptation phase consisted of 30 pointing trials with the thumb (10 for each target position) in alternation with 30 pointing trials with the index finger. Participants were wearing 10-diopter prism glasses over the shutter glasses. The prism in front of the left eye shifted the image of the target about 5 cm to the right. The prism in front of the right eye shifted the image of the target about 5 cm to the left. We opened the PLATO glasses in front of one eye at the start of each trial. In the first adaptation phase, we chose for each participant a correspondence between the viewing eye (and thus prism orientation) and the digit used. For the second adaptation phase, we switched which digit corresponded with which eye. Before each pointing trial, participants were told either to touch the left side of the target cube with their thumb or to touch the right side of the cube with their index finger. Once the shutter glasses in front of the corresponding eye opened, participants moved the appropriate digit to the appropriate side of the target cube.

### Data analysis

We analysed the hand movements in the two post-adaptation phases to determine the perceived size that the participant indicated. We defined it as the average distance between the markers on the index finger and thumb during the last 0.5 s of the 2 s of hand opening. We averaged these values over the three positions and five repetitions per object and phase. From the resulting value, we determined for each participant two measures to characterise their responses.

The first measure is the effect of object size: the difference in indicated size between the two object sizes. We expect this measure to be close to the actual size difference: about 2.5 cm. As this expectation is not hypothesis-driven, we do not test it formally. If the results would deviate considerably from the expected value, we would not be able to provide a reliable interpretation of the second measure.

The second measure is the one that can distinguish between the various hypotheses: the aftereffect, which we calculate as the difference between the indicated size in the post-adaptation phase after thumb-left/index-right adaptation and that after thumb-right/index-left adaptation. This approach differed slightly from the one we used in Schot et al. (2017): because we now performed both adaptations in the same session, we did not need to use the pre-adaptation phase as a reference. If grip aperture in grasping is based on perceived size (and the result of Schot et al. (2017) due to an effect of prism adaptation on size), this aftereffect should be larger than zero. More specifically, we expect it to be close to the 1.2 cm aftereffect that Schot et al. (2017) found in grip aperture. On the other hand, if grip aperture in grasping is based on perceived locations, we expect no aftereffect in the indicated size, and thus smaller than the value of Schot et al. (2017). We test both differences using one-sided *t* tests (using JASP): whether it is larger than zero a one-sample *t* test and whether it is smaller than the value of Schot et al. (2017) a two-sample *t* test.

## Results

**Fig. 2:**
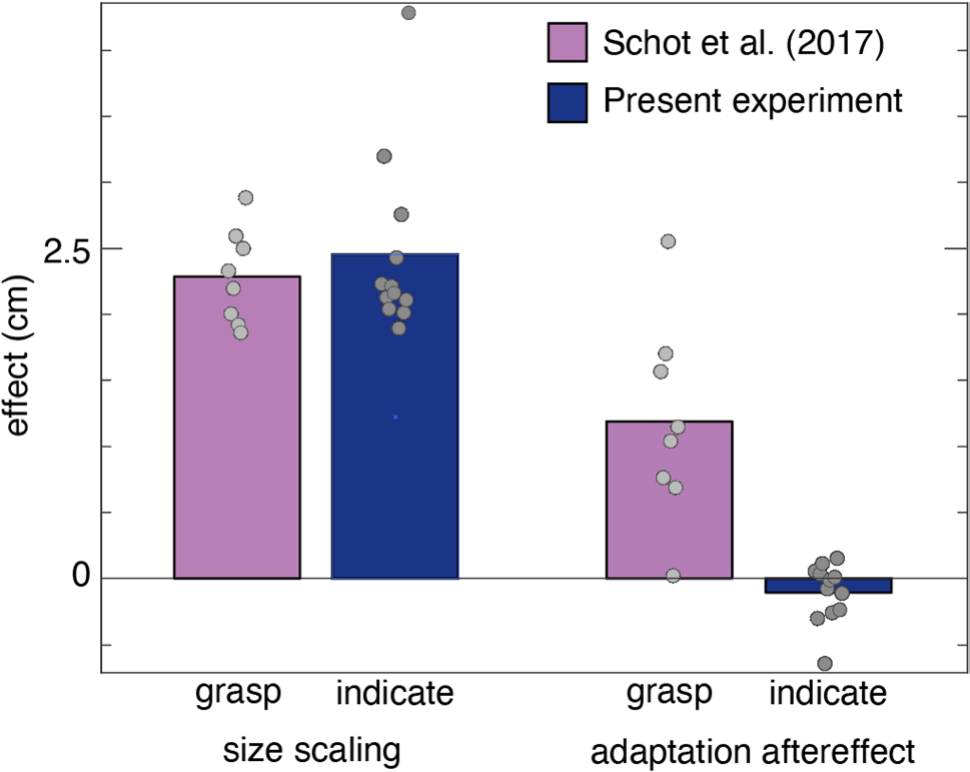
The results of the 12 participants in the indicating task of the present experiment, together with the results of the 8 participants in the grasping task (Schot et al. 2017). The ‘size scaling’ corresponds to the difference in grip aperture for the two object sizes. The ‘adaptation aftereffect’ corresponds to the difference in grip aperture between the two post-adaptation phases

Our participants were well able to indicate the perceived size of objects using their index finger and thumb. Their estimates of the cuboid and cube differed by 2.5 ± 0.7 cm (mean ± standard deviation across participants, left blue bar in Fig. 2), which corresponds well to the actual difference in size, and resembles the value we found previously for grip aperture in grasping (left pink bar in Fig. 2).

Our prime measure is the aftereffect: the difference in indicated size between the two post-adaptation sessions. We found an aftereffect of − 0.1 ± 0.2 cm (right blue bar in Fig. 2). In line with the negative sign, the one-sided one-sample *t* test showed that this aftereffect of adaptation is not larger than zero (*t*(11) = − 1.668, *p* = 0.938). The one-sided two-sample *t* test showed that it differed from that in grasping (right pink bar in Fig. 2 larger than the blue one; *t*(18) = 5.581, *p* < 0.001).

## Discussion

In this experiment, we showed that the independent visuomotor adaptation of the index finger and thumb that produces a clear aftereffect in grasping (Schot et al. 2017) does not affect the indication of perceived size using the finger and thumb. This finding provides clear evidence that grip aperture in grasping is not based on an estimate of object size. Before discussing the implications of this finding, we will first discuss whether the lack of an aftereffect might be caused by (one of) the two aspects in which the present perceptual experiment differed from the grasping experiment (Schot et al. 2017).

The two experiments differed in starting configuration: in the grasping experiment, each trial started with the hand holding a starting cube, whereas the hand was resting on the table in the present experiment. We introduced this change to prevent feeling the size of the starting cube at each trial to prevent adaptation of hand aperture. If this difference would have affected the results, it would have promoted (after-)effects of visuomotor adaptation in the present experiment and therefore cannot explain the lack of an aftereffect. The second difference is that the two opposing directions of adaptation were combined in a single session in the present experiment. Given the time course of adaptation (see Fig. 1A of Schot et al. 2017), we expect that the original adaptation has washed out completely after 90 trials. But even if this would be only partly the case, such a transfer of the previous adaptation could only cause a reduction, not a total abolishment of the aftereffect. Note that to correspond to the aftereffect on grasping, the aftereffect on indicating should be larger. The reason is that the magnitude of the aftereffect reported by Schot et al. (2017) is likely to be an underestimation, due to the way this after- effect was determined (Franz et al. 2001) and because grip aperture is limited on the positive side (Schenk et al. 2017).

The present results add to the interpretation of prism adaptation. Generally, prism adaptation is considered as consisting of two components: a slow automatic realignment between vision and proprioception (Salomonczyk et al. 2011; Tsay et al. 2022), combined with a deliberate strategic component (Kornheiser 1976; Prablanc et al. 2020). Any aftereffects are thus due to the realignment component. Our previous experiments on the adaptation of the index finger and thumb to opposite prism deviations (Schot et al. 2014, 2017) already severely limited the possibilities of sensory realignment. As the leftward and rightward displacements occurred at the same location in the visual field, the visual direction cannot be realigned. As the pointing movements of the index finger and thumb involve the same proximal muscles to transport the hand, any proprioceptive realignment must involve only muscles and joints that are distal to the wrist. To explain the aftereffects in pointing (Schot et al. 2014) and grip aperture in grasping (Schot et al. 2017) in terms of sensory realignment, one could argue that either visual size or proprioception of hand shape might be recalibrated. However, the fact that there is no aftereffect in grip aperture when indicating perceived size rules out this explanation. To understand what happens in prism adaptation, one should realise that matching locations is not just matching sensory representations (Kuling et al. 2017), but is based on sensory transformations that depend on the movement that needs to be made. This interpretation of adaptation strengthens the conclusion about the nature of grasping: the clear aftereffect on grip aperture in grasping (and not in indicating) indicates that grasping can be regarded as simultaneous one-digit pointing movements.

Our conclusion that the control of grasping is not based on perceived size is in sharp contrast with that of Franz and colleagues (Franz and Gegenfurtner 2008; Schenk et al. 2011; Kopiske et al. 2016). We have argued elsewhere that a very likely cause of this difference is that some size illusions such as the Ebbinghaus illusion also induce illusory position changes that are consistent with the changes in perceived size (Smeets and Brenner 2019; Smeets et al. 2020). In the present experiment, we used prism adaptation to induce changes in perceived position. We here show that these changes did not affect the perceived size. As our earlier experiment showed that this illusory position change resulted in a corresponding change in grip aperture (Schot et al. 2017), it is clear that grip aperture is not related to perceived size.

The dissociation between the perception of size and the control of grasping that we report seems to resemble a popular claim (Stöttinger and Perner 2006; Ganel et al. 2008a). However, our interpretation is different. The popular claim is formulated in terms of size processing: “Object size is processed differently for visually-guided action and for perception” (Ganel et al. 2008b p. R1090). In contrast, we claim that grip aperture is not related to any processing of size but is determined by the processing of location information. Formulated in the terminology of an early paper in the perception–action debate (Smeets and Brenner 1995), our claim is: “perception and action are based on the same visual information, but there is a distinction between position and size”. The only situation in which size information will be used in grasping is when contact positions are not visible, e.g. when grasping a cup of coffee in such a way that the contact point for the index finger is on the invisible back-side of the cup. In such situations, an estimate of object size can be used to estimate the location of the invisible contact point (Volcic and Domini 2014; Hesse et al. 2016b; Bozzacchi et al. 2018).

In summary: we showed that prism adaptation of movements of individual digits transfers to grasping, but not to the manual indication of perceived size. These results provide strong support for the digit-in-space hypothesis: grip aperture in grasping is controlled based on position information. Consequently, many experimental results that have been interpreted as a dissociation between perception and action are demonstrating a dissociation between size and position.

## Data Availability

The kinematics of all individual trials, the summary table and the Matlab code used are available at the OSF data repository (https://osf.io/cm8wy/).
